# Rare and Common Variants in *COL4A1* in Chinese Patients With Intracerebral Hemorrhage

**DOI:** 10.3389/fneur.2022.827165

**Published:** 2022-05-27

**Authors:** Xiaolu Liu, Qiong Yang, Lu Tang, Ji He, Danyang Tian, Baojun Wang, Lihong Xie, Changbao Li, Dongsheng Fan

**Affiliations:** ^1^Department of Neurology, Peking University Third Hospital, Beijing, China; ^2^Department of Neurology, Central Hospital of Baotou, Baotou, China; ^3^Department of Neurosurgery, Beijing Pinggu Hospital, Beijing, China; ^4^Beijing Municipal Key Laboratory of Biomarker and Translational Research in Neurodegenerative Diseases, Beijing, China; ^5^Key Laboratory for Neuroscience, National Health Commission/Ministry of Education, Peking University, Beijing, China

**Keywords:** Chinese, *COL4A1*, intracerebral hemorrhage, rare variants, single nucleotide polymorphism

## Abstract

Here, we screened the *COL4A1* variants in Chinese intracerebral hemorrhage (ICH) patients to summarize the relationship between the variants and clinical characteristics. Targeted sequencing of a 65-gene panel including *COL4A1* was performed to detect all the coding regions and ±10-bp splicing sites. In total, 568 patients were included. Regarding rare nonsynonymous variants with a minor allele frequency (MAF) <0.5%, 6 missense variants and five suspicious splice site variants, absent in 573 healthy controls, were found in 11 patients. The subgroup carrying rare variants did not show specific phenotype compared with non-variant carriers. For the single nucleotide polymorphism (SNP) loci with an MAF> 5%, we did not find a significant association between the allele or genotype distribution of the SNP loci and the risk of ICH. Rs3742207 was nominally associated with death at 1-year follow-up (*p* = 0.02027, OR 1.857, 95% CI 1.101-3.133) after adjusted by age, hypertension history, hematoma volume and recurrent ICH history. Nevertheless, after the Bonferroni correction, the association was no longer significant. In conclusion, rare nonsynonymous variants in *COL4A1* were identified in 1.94% (11/568) of Chinese ICH patients, while rs3742207 maybe indicate a worse prognosis of ICH.

## Introduction

China bears a large stroke burden worldwide, with an age-standardized prevalence of 1114.8/100,000 people and an incidence of 246.8/100,000 person-years in the latest epidemiologic survey in China (1). Intracerebral hemorrhage (ICH) accounts for 23.8% of incident strokes and 15.8% of prevalent strokes in China, and these values are significantly greater than those in high-income countries (9-13%) (2). Previous studies have suggested that genetic variation plays a substantial role in the occurrence and evolution of ICH in Caucasians (3). A systematic review and meta-analysis also showed that compared with being White, being Black or Hispanic was a risk factor for ICH (4). Genetic background is considered a determinant factor for the morbidity and clinical phenotype of ICH. Therefore, it is necessary to screen the risk genes, previously reported in Caucasians, in Chinese ICH patients.

*COL4A1* and *COL4A2*, the gene encoding type IV collagen α1 and α2, are located on chromosome 13 (13q34). Type IV collagen, the major component of the vascular basement membrane, regulates angiogenesis (5). Gould et al. first identified a French family carrying the G562E heterozygous mutation in *COL4A1* with multiple phenotypes of cerebral small vessel disease (CSVD), with ICH included (6). Subsequently, several studies have reported different phenotypes related to *COL4A1* rare mutations, including perinatal ICH, infantile hemiparesis, porencephaly, CSVD, epilepsy, retinal arteriolar tortuosity, congenital cataract, and hereditary angiopathy with nephropathy, aneurysm and cramps (HANAC) syndrome (7). *COL4A1* was eventually identified as a Mendelian gene of familial CSVD (8). In addition, patients with sporadic ICH have been reported to carry *COL4A1* rare mutations (9, 10). A high throughput sequencing (HTS) at 13q34 including *COL4A1* and *COL4A2*, performed in United States and Scotland based cohorts, identified rs138269346 (*COL*4*A*1^I110T^) and rs201716258 (*COL*4*A*2^H203L^) to be highly functional rare variants, and revealed that rs138269346 in *COL4A1* was nominally associated with non-lobar ICH risk (11). Another HTS identified 9 rare missense variants in *COL4A1* and *COL4A2* in MRI-confirmed CSVD, respectively (12). Common variants at 13q34, especially in *COL4A2*, were genetically associated with sporadic intracerebral hemorrhage (ICH), small vessel ischemic stroke and white matter hyperintensity based on genome-wide association studies (GWAS) conducted in individuals of European ancestry (13–19). Studies conducted in the cohort of 181 patients from Southwest China have focused on the common variants in *COL4A1*, indicated that the rs544012 AC genotype and rs679505 AA genotype were risk factors for ICH, while the rs532625 AA genotype was an independent adverse prognostic factor for ICH (20, 21). In addition, there are no studies about rare mutations of *COL4A1* in Chinese ICH patients. The purpose of this study was to screen for rare and common variants in the *COL4A1* gene and summarize the clinical characteristics related to *COL4A1* in a large Chinese population with primary ICH.

## Materials and Methods

### Subjects

In this multicenter cohort study, patients diagnosed with ICH by brain CT scans were prospectively recruited from 21 hospitals (Supplementary Table 1) in Beijing, Hebei and Inner Mongolia between 2015 and 2019. Patients with hemorrhage due to brain trauma, arteriovenous malformations, hemorrhagic tumors and hemorrhagic transformation after ischemic stroke were excluded. Demographic and clinical data were collected. Five hundred and sixty eight patients were recruited and prospectively followed up at 3 months, 6 months, and 1 year after admission.

Data from whole-exome sequencing conducted in 573 healthy subjects without cerebral vascular diseases were used as in-house controls. The study was approved by the Ethics Committee of each hospital [Institutional Review Board Number: (2014)医伦审第(191-2/3号)]. Written informed consent was obtained from each subject included in the study after the procedure was fully explained.

### Genetic Analysis

Genomic DNA was collected from peripheral blood leukocytes using a FlexiGene DNA Kit (Qiagen, Germany). A panel with 65 genes related to CSVD and cerebral amyloid angiopathy (Supplementary Table 2), including *COL4A1*, was designed to cover all the coding regions and ±10-bp splicing sites for these genes. DNA was enriched using the SureSelect Target Enrichment System (Agilent, US). High-throughput targeted sequencing was performed on a NEXTSEQ 500 device (Illumina, US).

Burrows Wheeler Aligner (BWA Version 0.7.15) was used for read alignment against the human reference genome hg19 (GRCh37). Quality control and variant calling, including single nucleotide variants (SNVs) and insertions and deletions (InDels), were carried out with the Genome Analysis Toolkit (GATK, Version 3.6). Annovar software (Version 2016-02-01) was used to annotate the variants against different public databases, including the Single Nucleotide Polymorphism (dbSNP; https://www.ncbi.nlm.nih.gov/snp), 1000 Genomes (http://browser.1000genomes.org), gnomAD v2.1.1 (https://gnomad.broadinstitute.org) and ChinaMAP (http://www.mbiobank.com/) databases. *In silico* tools, including SIFT (http://sift.jcvi.org), PolyPhen2 (http://genetics.bwh.harvard.edu/pph2/), and MutationTaster (http://www.mutationtaster.org), were used to predict the functional effects of the rare missense variants. For the function prediction of potential splice site rare variants, AdaBoost score and Random forests score in dbscSNV Splice Altering Predictions 1.1 were used (22). Functional annotation of common variants was based on RegulomeDB database v2.0.3 (http://regulome.stanford.edu), HaploReg v4.1 (http://pubs.broadinstitute.org/mammals/haploreg/haploreg.php) and Ensembl genome browser (http://www.ensembl.org/index.html).

### Statistical Analysis

Continuous variables that were abnormally distributed were expressed as medians (range) and compared using the Mann-Whitney *U*-test or Kruskal-Wallis test. Categorical variables were expressed as numbers (percentages) and analyzed using the Pearson χ2 test or Fisher's exact test. Significance was tested at the 5% level. A cutoff *p*-value ^*^n < 0.05 was considered statistically significant based on Bonferroni correction (n is the number of common variants). All analyses were performed using the SPSS Version 19 software package (IBM, US) and PLINK 1.9 (http://pngu.mgh.harvard.edu/purcell/plink/). To assess the burden of rare variants at the gene level, Combined Multivariate Collapsing (CMC) test and SNP-set kernel association test (SKAT-O) were conducted in Rvtest (23).

## Results

### Demographic and Clinical Characteristics

In total, 568 patients diagnosed with ICH with detailed clinical and genetic data were included in this study. The demographic and clinical characteristics of the ICH patients are shown in Table 1. Among them, 374 patients were male (65.7%). The median age of onset was 61 years (range 24–91). The median hematoma volume was 9.82 ml (range 0.04–159). With respect to hematoma location, lobar ICH (defined as lobar, cortical or cortico-subcortical hemorrhage) was present in 22.2% (126/568) of the patients, while non-lobar ICH (involving the putamen, thalamus, cerebellum, and brainstem) was present in 425 patients (74.8%). Another 17 patients (3.0%) suffered both lobar and non-lobar ICH. A total of 392 patients (69.0%) were diagnosed with hypertension. Eighty-two patients (14.4%) reported a history of ICH before this episode, and 65 patients (11.4%) died by the 1-year follow-up (Table 1).

**Table 1:** Demographic and clinical characteristics of ICH patients.

**Characteristics**	**Total ICH (*n* = 568)**	**ICH with rare variants in *COL4A1* absent in controls (*n* = 11)**	**ICH without rare variants in *COL4A1* absent in controls (*n* = 557)**	*P* value*
Age, median (range)	61 (24-91)	67 (43-88)	61 (24-91)	0.269
Male, *n* (%)	374 (65.8)	5 (45.5)	369 (66.2)	0.198
Hypertension, n (%)	392 (69.5)	6 (54.5)	386 (69.8)	0.468
Hematoma volume, median (range), ml	9.83 (0.04-159.02)	6.39 (0.5-159.02)	10 (0.04-151.78)	0.399
**Hematoma location**, ***n*** **(%)**				
Lobar	126 (22.2)	2 (18.2)	124 (22.3)	1.000
Non-lobar	425 (74.8)	9 (81.8)	416 (74.7)	
Both	17 (3.0)	0 (0)	17 (3.1)	
Recurrent intracerebral hemorrhage	82 (14.4)	3 (27.3)	79 (14.2)	0.203
Death at 1-year follow-up	65 (11.4)	3 (27.3)	62 (11.1)	0.121

*It was compared that the clinical manifestations of ICH patients with or without rare variants in COL4A1 absent in controls, and the results were listed in the Column “P-value”.

### Quality Control

The coverage of the target area in *COL4A1* was 100%. The average depth of sequencing in the target area in *COL4A1* was 100x. After excluding variants with sequencing depths <20x or heterozygosity <20%, we identified 3,706 nonsynonymous variants in the region of interest in *COL4A1*.

### Rare Variants

**Figure 1:**
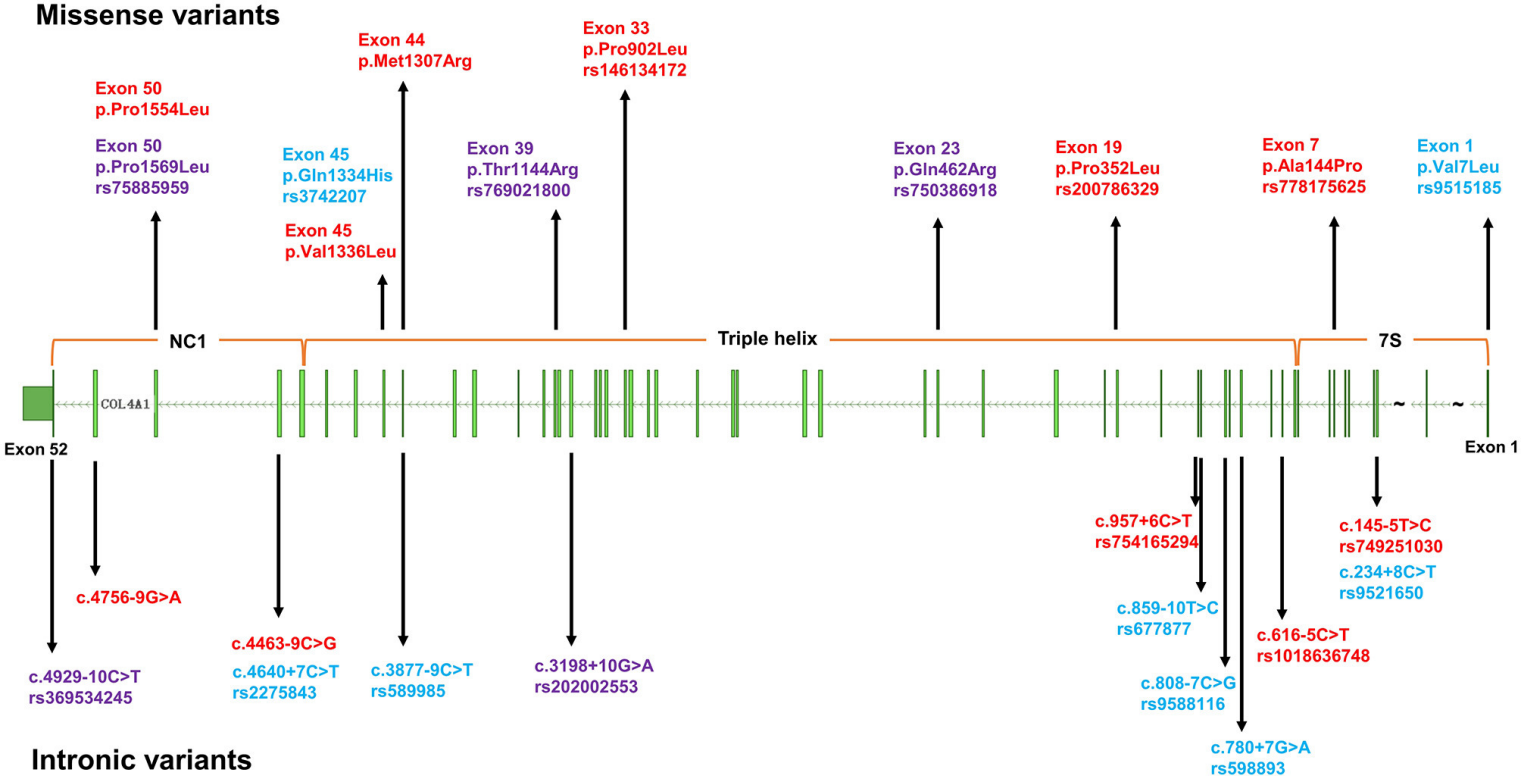
The distribution of variants in *COL4A1* detected in this study. The upper half of the figure depicts missense variants, the lower half intronic variants. Blue: common variants; red: rare variants only detected in cases; purple: rare variants detected in both cases and controls.

For the rare variants with a minor allele frequency (MAF) <0.5% in *COL4A1*, five variants were detected in both the ICH patients and controls (Supplementary Table 3). We also found another six missense variants and five suspicious splice site variants in *COL4A1* in 11 patients, and these variants were absent in 573 healthy controls. We focused on these 11 variants and speculated that they were more likely pathogenic mutations (Table 2; Figure 1). The frequency of these variants was <4^*^10^−5^ in gnomAD database and <3^*^10^−4^ in ChinaMAP database. None of these variants except Pro352Leu were reported in previous studies. Among the missense variants, Pro352Leu, Pro902Leu, Met1307Arg and Val1336Leu were located in the Triple helix domain, while Pro1554Leu and Ala144Pro were located in the NC1 and 7S domain, respectively. Surprisingly, the glycine residues were not involved. We conducted the burden analysis using variants with a MAF lower than 0.5% in the coding regions and ±10-bp in *COL4A1*. The result indicated that *COL4A1* was a risk gene for ICH (*p* = 3.75^*^10^−9^ in CMC, *p* = 4.1^*^10^−8^ in SKAT-O).

**Table 2:** List of rare variants in *COL4A1* identified in ICH patients, absent in controls, with related information in the public database and results predicted by *in silico* tools.

**CHROM: POS: REF: ALT (GRCh37)**	**Predicted consequences at the protein level**	**Domain**	**Frequency in patient allele**	**dbSNP**	**1000G_CHB**	**gnomAD**	**ChinaMAP**	**SIFT; Polyphen2; MutationTaster**	**Ada; RF**
13: 110804862: C: T	-	-	1/1136	N/A	N/A	None	None	N/A	0.000129436; 0.012
13: 110807724: G: A	p.Pro1554Leu	NC1	1/1136	N/A	N/A	None	A = 4.72233e^−05^	Deleterious; Probably damaging; disease_causing	N/A
13: 110813725: G: C	-	-	1/1136	N/A	N/A	None	None	N/A	0.00181727; 0.046
13: 110818594: C: G	p.Val1336Leu	Triple helix domain	1/1136	N/A	N/A	None	None	Tolerated; Benign; polymorphism	N/A
13: 110819534: A: C	p.Met1307Arg	Triple helix domain	1/1136	N/A	N/A	None	None	Tolerated; Benign; polymorphism	N/A
13: 110830200: G: A	p.Pro902Leu	Triple helix domain	1/1136	rs146134172	N/A	A = 0.00001415	None	Deleterious; Probably damaging; disease_causing	N/A
13: 110853814: G: A	p.Pro352Leu	Triple helix domain	1/1136	rs200786329	A = 0	A = 0.00004950	None	Deleterious; Probably damaging; disease_causing	N/A
13: 110857694: G: A	-	-	1/1136	rs754165294	N/A	A = 0.00001988	A = 0.00028334	N/A	2.4*10^−5^; 0
13: 110861779: G: A	-	-	1/1136	rs1018636748	N/A	A = 0.000004217	None	N/A	7.21*10^−6^; 0.006
13: 110864227: C: G	p.Ala144Pro	7S	1/1136	rs778175625	N/A	G = 0.000007954	G = 0.0000944465	Tolerated; Benign; polymorphism	N/A
13: 110866367: A: G	-	-	1/1136	rs749251030	N/A	G = 0.00003182	None	N/A	9.89*10^−5^; 0.02

*dbSNP, The Single Nucleotide Polymorphism Database; gnomAD, Genome Aggregation Database; ChinaMAP, China Metabolic Analytics Project; N/A, not available; Ada, AdaBoost score; RF, Random forests score*.

The details of clinical manifestations of these 11 patients were listed in Table 3. We found three patients reported an ICH history before this episode. Patient 1 (A2018012300201), with *APOE* ε2/ε2, suffered from lobar ICH 3 years before. In this course, we found the signals of hematoma, microbleeds and cortical superficial siderosis in lobes in the images of MRI. He experienced hematoma expansion twice and died 17 days after the onset. Patient 2 (C2015051301001), with *APOE* ε3/ε4, experienced an ICH in right cerebral hemisphere before. She suffered from cerebellar ICH with ventricular extension and the ventricular drainage was performed. Nevertheless, she died 3 months after the episode. Patient 3 (C2015113001101), with *APOE* ε3/ε3, suffered from ICH in left basal ganglia. It was found a stroke sac and lacunar infarcts in the basal ganglia with white matter hyperintensity in her CT scan. Although the ratio of recurrent ICH in patients with rare variants absent in controls was quite higher than the other group, the difference was not significant. There was also no significant difference in age of onset, hematoma volume and location, ratio of death by the 1-year follow-up between patients with or without the rare variants that were absent in the controls (Table 1). Porencephaly was not detected in the CT images of these 11 patients. White matter hyperintensities, cerebral microbleeds and lacunar infarction were found in five of the patients. We found mild arteriosclerosis in the retina instead of retinal arteriolar tortuosity in the patient with the p.V1336L variant (Supplementary Figure 1).

**Table 3:** Clinical information of the ICH patients with rare variants in *COL4A1* absent in controls.

**Patient ID**	**CHROM: POS: REF: ALT (GRCh37)**	**Gender**	**Age of onset**	**Hematoma location**	**Hematoma volume, ml**	**Hypertension**	**Recurrent intracerebral hemorrhage**	**Death at 1-year follow-up**	**CSVD in CT image**	**family history**	***APOE***
C2018080709301	13:110807724:G:A	Female	82	Basal ganglia	159.02	Yes	No	No	-	No	3/3
A2018012300201	13:110830200:G:A	Male	71	Lobar	7.52	No	Yes	Yes	Lobar CMB	No	2/2
A2018060500701	13:110818594:C:G	Female	64	Basal ganglia	4.53	No	No	No	-	No	3/3
C2015051301001	13:110866367:A:G	Female	55	Cerebellum	3.00	Yes	Yes	Yes	-	Yes	3/4
C2016072802001	13:110813725:G:C	Male	72	Basal ganglia	6.39	Yes	No	Yes	WMH	No	3/3
C2016072805701	13:110864227:C:G	Male	59	Basal ganglia	17.46	No	No	No	WMH, LI	Yes	3/4
C2015113001101	13:110819534:A:C	Female	75	Basal ganglia	0.50	Yes	Yes	No	WMH, LI	Not known	3/3
C2015101303301	13:110861779:G:A	Female	88	Lobar	0.92	No	No	No	-	No	3/3
C2019061304601	13:110804862:C:T	Male	43	Basal ganglia	16.91	No	No	No	LI	No	2/3
C2015101305401	13:110853814:G:A	Male	52	Basal ganglia	4.29	Yes	No	No	-	Not known	3/3
C2018051701501	13:110857694:G:A	Female	67	Basal ganglia	19.00	Yes	No	No	-	No	2/3

*ICH, Intracerebral hemorrhage; CSVD, cerebral small vessel disease; APOE, Apolipoprotein E; WMH, White matter hyperintensity; CMB, Cerebral microbleeds; LI, Lacunar infarcts; CT, computed tomography*.

### Common Variants

For the eight single nucleotide polymorphism (SNP) loci with an MAF > 5% (shown in Figure 1) in *COL4A1*, the frequency of genotype distribution in rs2275843 in controls did not accord with Hardy-Weinberg equilibrium (*P* < 0.001) (Supplementary Table 4). For the rest seven SNP loci, rs3742207 and rs9515185 were missense variants, the others were annotated intron or splice region variant. None of the seven SNP loci mapped to any known eQTL. Based on the RegulomeDB and HaploReg database, rs9515185 had the highest RegulomeDB score and was predicted to influence the motifs, protein bound and histone modification, indicating more likely to be a regulatory variant (Table 4).

**Table 4:** Functional annotation of common variants in *COL4A1*.

	**RegulomeDB rank**	**RegulomeDB score**	**Consequence ensembl**	**dbSNP functional annotation**	**Selected eQTL hits**	**Motifs changed**	**Proteins bound**	**Promoter histone marks**	**Enhancer histone marks**
rs9588116	4	0.60906	Splice region variant	Intronic	-	-	-	-	GI, LIV
rs3742207	5	0.13454	Missense variant	Missense	-	-	-	-	-
rs9521650	4	0.60906	Splice region variant	Intronic	-	SEF-1	-	-	9 tissues
rs9515185	4	0.70497	Missense variant	Missense	-	5 altered motifs	POL2, CTCF, ZNF263	23 tissues	BLD
rs589985	5	0.13454	Intron variant	Intronic	-	-	-	-	SKIN
rs598893	5	0.13454	Splice region variant	Intronic	-	Egr-1,TBX5	-	-	GI, KID
rs677877	5	0.13454	Intron variant	Intronic	-	Pax-4,Pou5f1	-	-	GI

In the seven SNP loci in *COL4A1*, no significant difference was found in the allele distribution in ICH patients compared with the controls. Genotypic distribution was not considered to be related with the ICH risk in the analysis of codominant model, dominant model, or recessive model (Supplementary Tables 4, 5).

Then, we analyzed the association between the alleles of the seven SNP loci in *COL4A1* and the prognosis of ICH (death by the 1-year follow-up). In the logistic regression test, rs3742207 was nominally associated with death at 1-year follow-up (*p* = 0.02027, OR 1.857, 95% CI 1.101-3.133) after adjusted by age, hypertension history, hematoma volume and recurrent ICH history (Table 5). Nevertheless, after the Bonferroni correction, the association was no longer significant (*p* > 0.0071). In the subgroup analysis in regard to with or without recurrent ICH history, lobar or non-lobar ICH and with or without family history, there was a trend but not a significant association after the Bonferroni correction between rs9515185 and death at 1-year follow-up in the subgroup without recurrent ICH history, non-lobar ICH and without family history (Supplementary Table 6). In the codominant model, dominant model, or recessive model, the genotypic distribution was not significantly related with the death at 1-year follow-up. In Kaplan-Meier survival analysis, no significant difference was found in the genotypic distribution of the seven SNPs in the codominant model, dominant model, or recessive model (Supplementary Table 7).

**Table 5:** Association between alleles of *COL4A1* and the death at 1-year follow-up in intracerebral hemorrhage patients.

**SNP**	**Effect allele**	**MAF in Death**	**MAF in Survival**	***P*-value**	**Adjusted OR (95% CI)**	**Adjusted *P*-value**
rs9588116	C	0.2623	0.2299	0.4258	0.8695 (0.5426, 1.393)	0.5612
rs3742207	G	0.1803	0.2617	0.05164	1.857 (1.101, 3.133)	**0.02027**
rs9521650	A	0.2787	0.2881	0.8282	1.134 (0.7157, 1.797)	0.592
rs9515185	C	0.2951	0.3814	0.06348	1.363 (0.8924, 2.081)	0.1518
rs589985	G	0.2705	0.3411	0.1194	1.529 (0.9806, 2.384)	0.06097
rs598893	C	0.2623	0.2331	0.4743	0.9022 (0.5634, 1.445)	0.6684
rs677877	A	0.2623	0.2309	0.4417	0.8764 (0.5467, 1.405)	0.5838

*MAF, Minor allele frequency; Adjusted by age, hypertension history, hematoma volume and recurrent ICH history. Bold value means rs3742207 was nominally associated with death at 1-year follow-up*.

## Discussion

In the present study, we screened rare and common variants in *COL4A1* by HTS in 568 ICH patients from Northern China. In total, we found that 11 ICH patients carried 11 rare nonsynonymous variants absent in controls. Common variants were not significantly associated with the risk or prognosis of ICH, while rs3742207 was nominally associated with death at 1-year follow-up. Previously, studies on rare or common variants in *COL4A1* were conducted in ICH or CSVD patients, mostly in populations of European ancestry. Another study focusing on this topic, conducted in Southwest China, investigated the association between 6 SNPs and the risk or prognosis of ICH (20, 21). Therefore, the present study comprises the largest number of patients to date for the comprehensive exploration of the role of *COL4A1* variants in ICH in Chinese patients.

*COL4A1* and *COL4A2*, encoding the α1 and α2 chains, are connected by a common 130-base pair promoter region. Each chain consists of 3 domains: (1) a short N-terminal 7S domain, (2) a long triple helical collagenous domain containing the classic G-X-Y repeat amino acid sequence, and (3) a C-terminal noncollagenous NC1 domain rich in cysteine and lysine. The secretion of mutated *COL4A1* is impaired, and its accumulation results in focal disruption of the basement membrane (5). A recent study sequencing at 13q34 with *COL4A1* and *COL4A2* included, identified two rare SNPs to be highly functional rare variants in sporadic ICH. The *in silico* model predicted the mutants altered physical length and thermal stability of collagen (11). Most of the rare mutations previously reported in *COL4A1* have been concentrated in highly conserved glycine residues, which result in typical and severe clinical manifestations. Nevertheless, in the present study, we did not find glycine missense mutations. Pro352Leu and Pro902Leu both affected the Y position of the triple helix domain. During collagen biosynthesis, prolines are converted to hydroxyprolines, which are critical for cross-linking of collagen heterotrimers (24). Pro352Leu was previously reported in a sporadic ICH patient who was diagnosed with possible cerebral amyloid angiopathy. Functional tests confirmed that the mutation Pro352Leu resulted in reduced extracellular secretion and intracellular aggregation (9). This variant was also reported in a patient with history of hypertension and migraine with aura, whose MRI imaging features included multiple lacunar infarcts, mild white matter hyperintensities, and no microbleeds (12). In the present study, the patient with Pro352Leu had a different phenotype and was more likely to have hypertensive deep ICH with an age of onset of 52 years. This finding supported that *COL4A1* disorder has a variable phenotype even in a family with the same mutation. Ala144Val was found in a control cohort and did not affect extracellular secretion *in vitro* (9). Ala144Pro was located in the same site in the 7S domain, and further functional verification is needed to determine whether this mutation affects molecular crosslinking. Other reported mutations located in another two domains or non-G in the triple helical collagenous domain, such as Lys950Glu, Ser1582Pro, and Met1Thr, usually indicate milder phenotypes (5). A study of mouse model also showed that the mutation in the Yaa residue of the collagen caused a milder retinal appearance (25). Based on this phenomenon, we hypothesized that Met1307Arg, Val1336Leu and Pro1554Leu caused minor functional impairment. In addition to missense mutations, we also found 5 splicing region variants. *In silico* analysis of these five variants didn't show a significant potential to affect splicing. Previously reported splicing or frameshift mutations, c.2085del and c.2194-1G > A, lead to haploinsufficiency, which may be less damaging than dominant-negative effects (26, 27). In terms of the clinical features of ICH, not all patients present with deep hemorrhage. Some patients with lobar hemorrhage might also suffer cerebral amyloid angiopathy under the influence of other genes. We did not identify porencephaly or retinal arteriolar tortuosity in our patients. These characteristics seem different from those of typical glycine missense mutation-related ICH.

In recent years, several studies have focused on the relationship between common variants in *COL4A1*/*COL4A2* and sporadic ICH. Studies conducted in individuals of European ancestry have identified the genetic loci at 13q34 as having genome-wide significant association with ICH. Several SNPs at *COL4A2*, such as rs9515201, have been associated with deep or non-lobar ICH (13, 17, 19). Two studies conducted in Southwest China analyzed the relationship between six SNPs in *COL4A1* and the risk and outcome of ICH (20, 21). Only rs3742207 in the above two studies overlap with the present study. It was shown a trend but not a significant tendency that rs3742207 increased the risk of death at 1-year follow-up in the present study. While in the previous study, rs3742207 was not related with death or disability at 3-month or 6-month follow-up. The sample size and coverage of loci in these three studies have been limited, and these findings merit replication studies such as GWAS.

Our study had several limitations. First, it is unfortunate that *COL4A2* was not included in the gene panel; therefore, we could not analyze mutations in these two closely related genes, namely, *COL4A1* and *COL4A2*, simultaneously. Second, the gene panel targeted only the coding regions and flanking 10bp, so variants in other regions were not included. In addition, copy number variants were not accurately analyzed. Third, the case report form was not specially designed for *COL4A1* syndrome, so a mass of data related to multisystemic phenotypes, such as muscle cramps, epilepsy, and HANAC syndrome, were not collected. Some important information, such as the family history of 73 of the ICH patients and the ages of the controls, was not available. Finally, we did not complete functional tests to determine whether the variants affected protein secretion and basal membrane function.

In summary, rare nonsynonymous variants in *COL4A1* were detected in 1.94% (11/568) of Chinese ICH patients. Common variants in *COL4A1* were not found to be associated with the risk of ICH, while rs3742207 maybe nominally associated with death at 1-year follow-up.

## Data Availability Statement

The datasets presented in this study can be found in online repositories. The name of the repository and accession number can be found below: National Center for Biotechnology Information (NCBI) BioProject, https://www.ncbi.nlm.nih.gov/bioproject/, PRJNA831362.

## Ethics Statement

The studies involving human participants were reviewed and approved by Medical Scientific Research Ethics Committee Peking University Third Hospital. The patients/participants provided their written informed consent to participate in this study. Written informed consent was obtained from the individual(s) for the publication of any potentially identifiable images or data included in this article.

## Author Contributions

XL: conceptualization, investigation, formal analysis, and writing—original draft. QY: investigation, project administration, and writing—review. LT: investigation, methodology, formal analysis, validation, and writing—review and editing. JH: methodology, formal analysis, and writing—review and editing. DT: investigation, methodology, and formal analysis. BW, LX, and CL: resources and validation. DF: conceptualization, writing—review and editing, supervision, and funding acquisition. All authors contributed to the article and approved the submitted version.

## Funding

This study was supported by Beijing Municipal Science and Technology Commission (grant number D141100000114005) and National Natural Science Foundation of China (grant numbers 81471184, 81901298, and 81901204).

## Conflict of Interest

The authors declare that the research was conducted in the absence of any commercial or financial relationships that could be construed as a potential conflict of interest.

## Publisher's Note

All claims expressed in this article are solely those of the authors and do not necessarily represent those of their affiliated organizations, or those of the publisher, the editors and the reviewers. Any product that may be evaluated in this article, or claim that may be made by its manufacturer, is not guaranteed or endorsed by the publisher.

## Abbreviations

CSVD: cerebral small vessel disease
CT: computed tomography
gnomAD: Genome Aggregation Databases
GWAS: genome-wide association study
HANAC: hereditary angiopathy with nephropathy, aneurysm and cramps
ICH: intracerebral hemorrhage
InDel: insertion and deletion
MAF: minor allele frequency
SNP: single nucleotide polymorphism
SNV: single nucleotide variant.
