# Supplementary material for: Rare and Common Variants in COL4A1 in Chinese Patients With Intracerebral Hemorrhage
Source: Front Neurol. 2022 May 27;13:827165. doi: 10.3389/fneur.2022.827165 (PMC9196627; doi:10.3389/fneur.2022.827165)

Supplementary Table 1. List of the 21 hospitals included in this study

01- Peking University Third Hospital
02- Beijing Tiantan Hospital
03- Beijing Shijitan Hospital
04- Beijing Daxing People's Hospital
05- Aviation General Hospital
06- Beijing Haidian Hospital
07- Beijing Liangxiang Hospital
08- 263 Hospital of the People's Liberation Army
09- Aerospace Centre Hospital
10- Beijing Renhe Hospital
11- Beijing Pinggu Hospital
12- General Hospital of Beijing Military Command
13- Beijing Huairou Hospital
14- Beijing Tongzhou Luhe Hospital
15- Beijing Fuxing Hospital
16- Beijing Friendship Hospital
17- Beijing Shougang Hospital
18- Beijing Yanqing Hospital
19- Hebei Kailuan General Hospital
20- Hebei Handan People's Hospital
21- Central Hospital of Baotou, Inner Mongolia

Supplementary Table 2. Gene list of the target sequencing panel.

A2M	C1D	GLA	ITM2B	PSEN2
ABCA1	C1R	GSN	LRP1	RPS27A
ABCB1	CAV1	GSTO1	LTBP1	RPSA
ABCC6	CAV2	HSPB2	MAPT	SERPINA3
ABCG2	CH25H	HSPB6	MBP	SORL1
ABRA	COL4A1	HSPB8	MME	TGFB1
ACE	COLEC12	HTRA1	MMP9	TOMM40
AOC3	CR1	IDE	NOTCH3	TREX1
APOE	CST3	IFNAR1	OLR1	TTR
APP	CTSD	IL1A	PLAT	TUBA1B
AQP1	CYP46A1	IL1B	PON1	TUBB
AQP4	DDR1	IL33	PRNP	TUBB4A
BCHE	ERCC6L	IL6	PSEN1	VEGFA

Supplementary Table 3. List of rare variants in *COL4A1* identified in both ICH patients and in-house controls with related information in the public database and results predicted by *in silico* tools.

CHROM:POS: REF:ALT (GRCh37)	Predicted consequences at the protein level	domain	Frequency in patient allele	Frequency in control allele	p value	Odds ratio (vs. in-house controls)	dbSNP	1000G_CHB	gnomAD East Asian	ChinaMAP	SIFT; Polyphen2; MutationTaster	Ada; RF
13:110802801: G:A	N/A	-	1/1136	1/1146	1.000	1.009 (0.06-16.1）	rs369534245	N/A	A=0.00005017	A=9.44465e-05	N/A	0.000152609; 0.012
13:110807679: G:A	p.Pro1569Leu	NC1	1/1136	2/1146	1.000	0.504 (0.04-5.56)	rs75885959 (EAS=0.004)	A=0.0049	A=0.0007518	A=0.00108614	Tolerated; Benign; disease_causing	N/A
13:110826321: G:C	p.Thr1144Arg	Triple helix domain	2/1136	1/1146	0.623	2.019 (0.18-22.3)	rs769021800	N/A	C=0.000752	C=0.00179448	Tolerated; Possibly damaging; polymorphism	N/A
13:110827555: C:T	N/A	-	1/1136	2/1146	1.000	0.504 (0.04-5.56)	rs202002553 (EAS=0.001)	T=0	T=0.003608	T=0.00132225	N/A	N/A
13:110845257: T:C	p.Gln462Arg	Triple helix domain	1/1136	1/1146	1.000	1.009 (0.06-16.1）	rs750386918	N/A	C=0.0002718	C=0.00028334	Tolerated; Benign; disease_causing	N/A

Key: dbSNP: The Single Nucleotide Polymorphism Database; gnomAD: Genome Aggregation Database; ChinaMAP: China Metabolic Analytics Project; N/A, not available; Ada, AdaBoost score; RF, Random forests score.

Supplementary Table 4. Association between alleles of *COL4A1* and the risk of intracerebral hemorrhage.

SNP	Effect allele	MAF in ICH	MAF in Control	P value	HWE in control P value
rs9588116	C	0.2342	0.2382	0.8192	0.818
rs3742207	G	0.25	0.2408	0.6111	0.01616
rs9521650	A	0.2879	0.2792	0.6478	0.7566
**rs2275843**	A	0.3028	0.3037	0.9649	**0.000776**
rs9515185	C	0.3732	0.3517	0.2836	0.1997
rs589985	G	0.3292	0.3421	0.5162	0.001117
rs598893	C	0.2368	0.2592	0.2161	0.2319
rs677877	A	0.235	0.2531	0.3164	0.5069

Key: MAF: Minor allele frequency; ICH, Intracerebral hemorrhage; HWE: Hardy-Weinberg equilibrium.

Supplementary Table 5. Association between genotypes of *COL4A1* and the risk of intracerebral hemorrhage.

SNP	Genotype	ICH	Control	Codominant model P value	Dominant model P value	Recessive model P value
rs9588116	CC	33	31	0.8357	0.6778	0.7692
	CG	200	211			
	GG	335	331			
rs3742207	GG	41	44	0.6154	0.4321	0.767
	GT	202	188			
	TT	325	341			
rs9521650	AA	48	46	0.9002	0.6601	0.7951
	AG	231	228			
	GG	289	299			
rs9515185	GG	240	248	0.2951	0.7258	0.1223
	GC	232	247			
	CC	96	78			
rs589985	AA	265	266	0.4827	0.9372	0.2512
	AG	232	222			
	GG	71	85			
rs598893	TT	332	320	0.395	0.3741	0.2083
	TC	203	209			
	CC	33	44			
rs677877	GG	334	323	0.5973	0.4058	0.419
	GA	201	210			
	AA	33	40			

Key: ICH, Intracerebral hemorrhage

Supplementary Table 6. Subgroup-analysis between alleles of *COL4A1* and the death at 1-year follow-up in intracerebral hemorrhage patients.

SNP	Effect allele	P value		P value		P value	
		With recurrent ICH history	Without recurrent ICH history	Lobar ICH	Non-lobar ICH	With family history	Without family history
rs9588116	C	0.8644	0.302	0.2148	0.9817	0.4742	0.5339
rs3742207	G	0.3217	0.1687	0.5587	0.1537	NA	0.7193
rs9521650	A	0.9268	0.6806	0.1209	0.5417	0.4925	0.6877
rs9515185	C	0.8733	**0.0385**	0.6527	**0.01524**	0.1849	**0.0285**
rs589985	G	0.2856	0.4899	0.3119	0.5486	0.1441	0.9386
rs598893	C	0.6809	0.3127	0.2148	0.915	0.4742	0.5893
rs677877	A	0.8644	0.3127	0.2148	0.9594	0.4742	0.5521

Key: ICH, Intracerebral hemorrhage.

Supplementary Table 7. Association between genotypes of *COL4A1* and the death at 1-year follow-up in intracerebral hemorrhage patients.

SNP	Genotype	Death	Survival	P value in Fisher's exact test			P value in Kaplan-Meier estimator		
				Codominant model	Dominant model	Recessive model	Codominant model	Dominant model	Recessive model
rs9588116	CC	4	28	0.6245	0.4076	0.7762	0.577	0.298	0.85
	CG	24	161						
	GG	33	283						
rs3742207	GG	1	36	0.1272	0.1337	0.1063	0.182	0.393	0.068
	GT	20	175						
	TT	40	261						
rs9521650	AA	4	39	0.9566	1	0.8054	0.798	0.976	0.517
	AG	26	194						
	GG	31	239						
rs9515185	GG	7	82	0.2227	0.09954	0.2791	0.199	0.296	0.08
	GC	22	196						
	CC	32	194						
rs589985	AA	5	61	0.3651	0.1744	0.4078	0.473	0.225	0.562
	AG	23	200						
	GG	33	211						
rs598893	TT	4	28	0.6947	0.4901	0.7762	0.643	0.832	0.348
	TC	24	164						
	CC	33	280						
rs677877	GG	4	28	0.6587	0.4093	0.7762	0.597	0.85	0.313
	GA	24	162						
	AA	33	282						

Supplementary Figure 1. The images of the retina in the patient with p.V1336L variant in *COL4A1*.

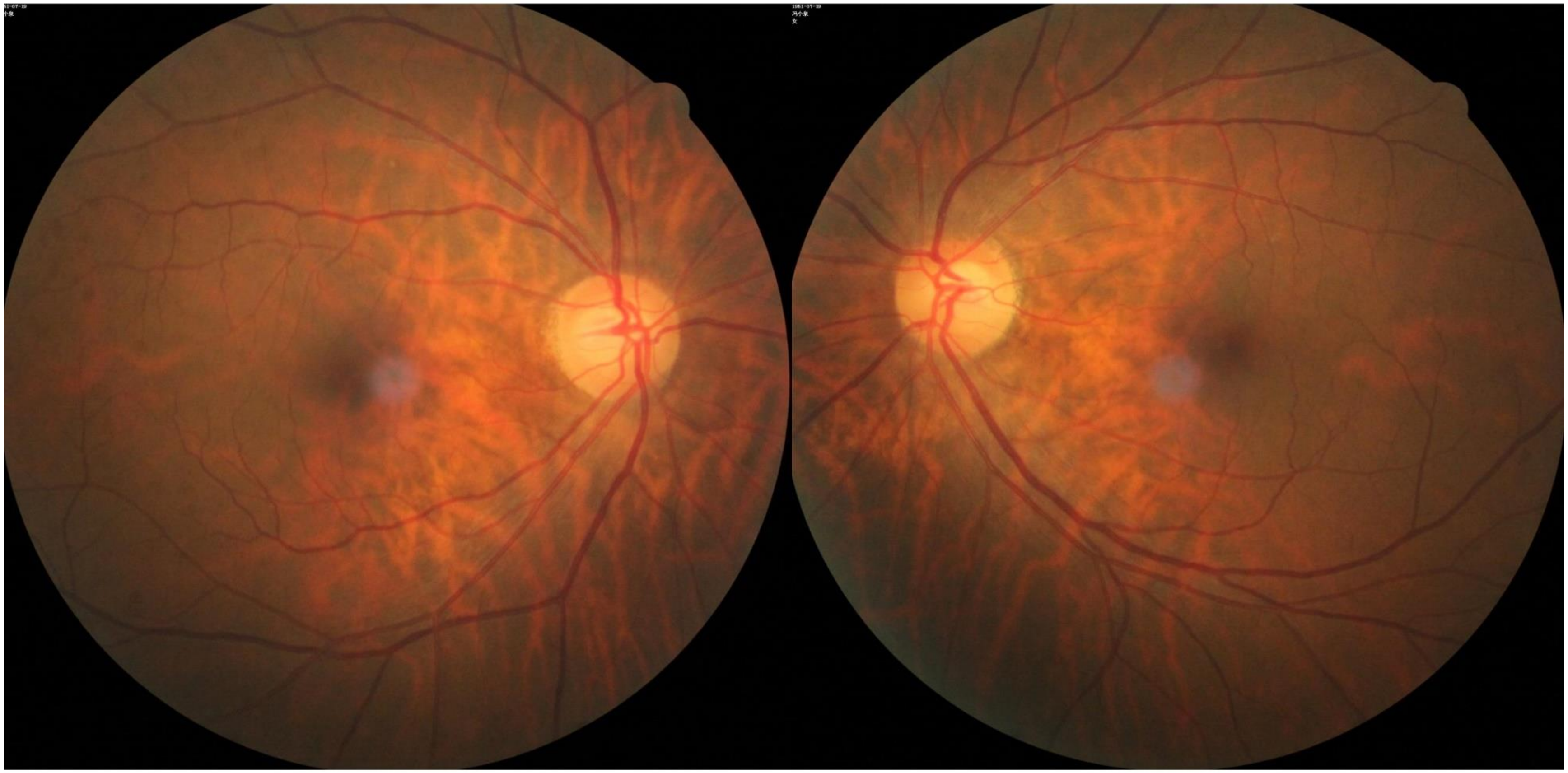

Supplement: Supplementary file 1 [file Data_Sheet_1.pdf]
